# Identification of The Fipronil Resistance Associated Mutations in *Nilaparvata lugens* GABA Receptors by Molecular Modeling

**DOI:** 10.3390/molecules24224116

**Published:** 2019-11-14

**Authors:** Yafeng Tian, Ya Gao, Yanming Chen, Genyan Liu, Xiulian Ju

**Affiliations:** Key Laboratory for Green Chemical Process of Ministry of Education, School of Chemical Engineering and Pharmacy, Wuhan Institute of Technology, Wuhan 430205, China; 21706010103@wit.edu.cn (Y.T.); gaoya_007@yeah.net (Y.G.); yanmingchen2018@163.com (Y.C.)

**Keywords:** fipronil, resistance, homology modeling, GABA receptors, RDL

## Abstract

Fipronil, as the first commercialized member of phenylpyrazole insecticides, has been widely used to control planthoppers in China due to its high insecticidal activity and low toxicity to mammals. However, insects have developed resistance to phenylpyrazoles after their long-term use. The resistance mechanism of insects to fipronil has not been well identified, which limited the development of phenylpyrazole insecticides. In the present study, we aimed to elucidate the related fipronil-resistance mechanism in *N. lugens* GABA receptors by homology modeling, molecular docking, and molecular dynamics. The results indicated that fipronil showed the weakest interaction with the mutant (R0′Q + A2′S) GABA receptors, which is consistent with the experimental study. The binding poses of fipronil were found to be changed when mutations were conducted. These findings verified the novel fipronil-resistance mechanism in silico and provide important information for the design of novel GABAR-targeting insecticides.

## 1. Introduction

Fipronil is the first generation commercialized phenylpyrazole (fiprole) insecticide that has been widely applied in agricultural pest control with its high insecticidal activity and good selectivity to mammals. Fipronil was considered to have better activity when compared with traditional insecticides, such as dieldrin [1] and dichlorodiphenyltrichloroethane [2]. The bioactivity of fipronil is ascribed to its ability to target ionotropic γ-aminobutyric acid (GABA) receptors (GABARs) [3]. Fipronil plays the role of the noncompetitive blocker of the GABAR in the central nervous system (CNS) of insects.

The ionotropic GABAR is a member of the pentameric transmembrane cys-loop ligand-gated ion channel family mediating synapse inhibition in insect CNS, and the GABAR is also one of the most significant insecticide targets [4]. Similar to other neurotransmitter-gated ion channels, the GABAR contains a central chloride ion channel pore jointly assembled by five transmembrane subunits. To date, 19 subunits from eight different subtypes (α_1−6_, β_1−3_, γ_1−3_, δ_1_, ε_1_, θ_1_, π_1_, and ρ_1−3_) have been identified in mammals. Each subunit consists of three well-defined domains: the extracellular domain, the transmembrane domain (TM1–4) formed by four α-helix segments, and a cytoplasmic loop of variable length between TM3 and TM4 α-helices [5]. The five TM2 domains constitute the ion channel, which allows for chloride ions to be transferred from outside the cell to the intracellular compartment. Among these domains, TM2s are believed to be the binding site of traditional noncompetitive antagonists (NCAs), such as fipronil. The residues in the TM2 membrane-spanning region are designated with an index numbering system to compare TM2 mutations of GABARs in different species. According to the number system, the residue arginine at the cytoplasmic end of TM2 is numbered 0′ [6].

In insect GABARs, fipronil binds to the chloride channel of TM2 domains and act as an NCA, which is able to block the normal transfer of chloride ions. At the same time, the normal function of the CNS will also be disturbed, which leads to insect overexcitement and convulsions until death at last. Although fipronil has displayed superiority when comparing to traditional pesticides, its high toxicity to fishes and honeybees limit the use of fipronil. Furthermore, resistance problems are a major obstacle in the application of fipronil [7]. At present, three major insecticide resistance mechanisms have been identified: one is the metabolic detoxification through the overexpression of metabolic genes and another is the reduce penetration or increase excretion [8]. Apart from these mechanisms, the resistance also acts as a result of amino acid mutations in the target site of insecticides. For example, Hope et al. reported that the GABAR T6′L mutation has an effect on dieldrin resistance in cattle tick, *Rhipicephalus (Boophilus) microplus* [9]. Torres et al. observed a leucine-to-phenylalanine mutation in GABAR transmembrane segment IIS6 that played an important role in the resistance of DDT and pyrethroid insecticides in peach-potato aphid, *Myzus persicae* [10].

The insect RDL (resistant to dieldrin) GABAR (RDLR) is a significant target of insecticides. The insect RDL subunit gene was cloned from dieldrin-resistant *Drosophila melanogaster* and named as *Rdl* [11]. Previous studies have suggested that the A2′S mutation located at the TM2 domain of RDL has crucial influence on insecticides resistance, such as dieldrin and fipronil [12,13,14,15,16,17,18]. Recently, Zhang et al. revealed that the mutation R0′Q in *Nilaparvata lugens* RDL in combination with A2′S is associated with much higher levels of resistance [19].

In this paper, computer-aided methods involving protein homology modeling, molecular docking, and molecular dynamics (MD) simulations were applied to explore the interaction modes of fipronil in wild-type and mutant *N. lugens* RDLRs to further validate and study the fipronil-resistance mechanism. Computer-aided methods become more and more significant and it has exerted important implications for the molecular design and mechanism explanation [20,21,22,23,24]. In this paper, three different comparative methods were applied to construct the three-dimensional (3D) structure of *N. lugens* RDLRs, and the best one was then chosen to generate mutant models, including A2′S, R0′Q, and dual-mutation A2′S + R0′Q. Fipronil were docked into these constructed models, and the fipronil-RDLR complexes were conducted for 20 ns MD simulation. The binding free energies and binding pattern of fipronil in *N. lugens* RDLRs were further analyzed. The results verified the previously experimental data and may contribute to the understanding of fipronil-resistance mechanism in insect RDLRs and the development of novel insecticides.

## 2. Results and Discussion

### 2.1. Homology Model

**Figure 1:**
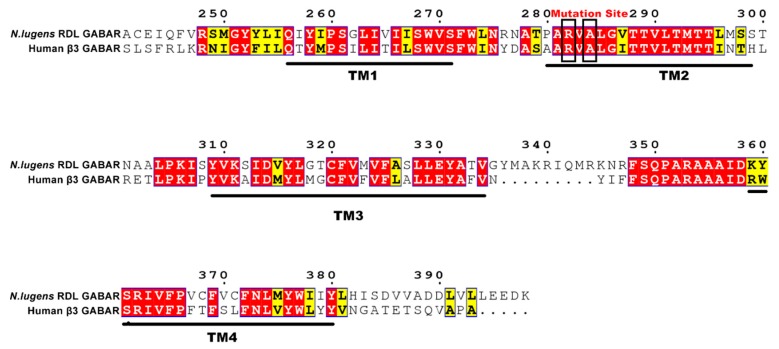
Sequence alignment of four transmembrane (TM) domains between *N. lugens* Resistant to dieldrin (RDL) and Human GABAR β3 subunits. The mutation sites are marked by black frames.

As for homology modeling, different factors, such as template selection and alignment accuracy, might have an important effect on the accuracy of the model. Owing to the absence of the crystal structure of insect RDLRs, the crystal structure of Human β3 GABA_A_R (PDB ID: 4COF) was chosen as the template for homology modeling of *N. lugens* RDLR. As the template, GABA_A_R β3 subunit possesses good homology to RDL subunit. In addition, Human β3 GABA_A_R and insect RDLR are both homologous pentamers. Thus, the crystal structure of Human β3 GABA_A_R was suitable to act as the template for homology modeling. Figure 1 illustrated that the *N. lugens* RDL subunit is highly homologous to Human GABAR β3 subunit with a 40.65% sequence identity, especially in the fipronil target site of TM2 domain. The multiple sequence alignment (MSA) was carried out using ClustalW.

**Figure 2:**
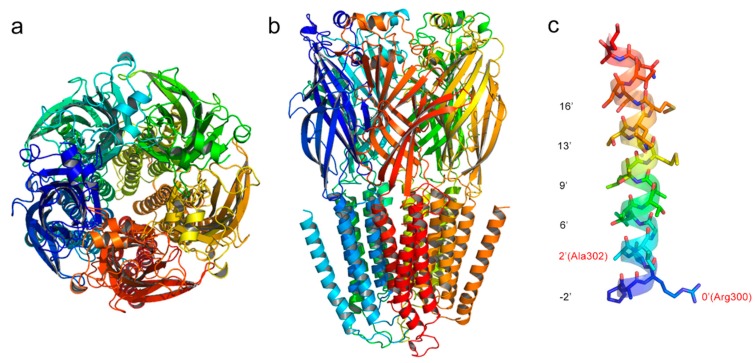
The *N. lugens* RDL GABA receptors (RDLR) model constructed by SWISS-MODEL. (**a**) side view, (**b**) top view, and (**c**) TM2 domain. The residues are designated with an index numbering system for TM2 membrane-spanning region, and the mutant sites are colored in red.

The three-dimensional (3D) model of *N. lugens* RDLR was constructed using three comparative tools. All of the constructed models were then evaluated to choose the best one. Table 1 shows the evaluation results. The quality of the generated models was evaluated by the Ramachandran plot that was acquired from PROCHECK program. Generally, a good quality model is preferred to have more than 90% of the residues in the favored regions of the Ramachandran plot [25]. Table 1 demonstrated that 99.5% residues of the model that were generated by SWISS-MODEL were confined to allowed regions, and only 0.5% residues were in disallowed regions. Figure S1 shows the Ramachandran plot of the constructed *N. lugens* RDLR model. These parameters are close to those of the other two models. The overall quality factor evaluated by ERRAT always have a proportional relationship with the quality of model. The score of SWISS-MODEL-modeled structure is much higher than those of the other models. Furthermore, the Z-score of the SWISS-MODEL-generated model is slightly higher than those of the other models. Table S1 shows the evaluation assessment of template and mutant *N. lugens* RDLR models. Consequently, the *N. lugens* RDLR model that was generated by SWISS-MODEL was considered to be the best model and it was used for investigating the fipronil-resistance mechanism through molecular docking and MD simulation. Figure 2a,b show the 3D structure of the best model that was constructed by SWISS-MODEL. The residues in the TM2 helices were renumbered while using an index numbering system to recognize the corresponding positions as shown in Figure 2c [6].

### 2.2. Model Optimization

20 ns MD simulations were performed on wild-type, A2′S, R0′Q, and dual-mutation (A2′S + R0′Q) models, respectively, to obtain stable structures. After MD simulations, potential energy (PE), radius of gyration (Rg), and root mean square deviation (RMSD) were calculated to assess whether the structure is stable.

**Figure 3:**
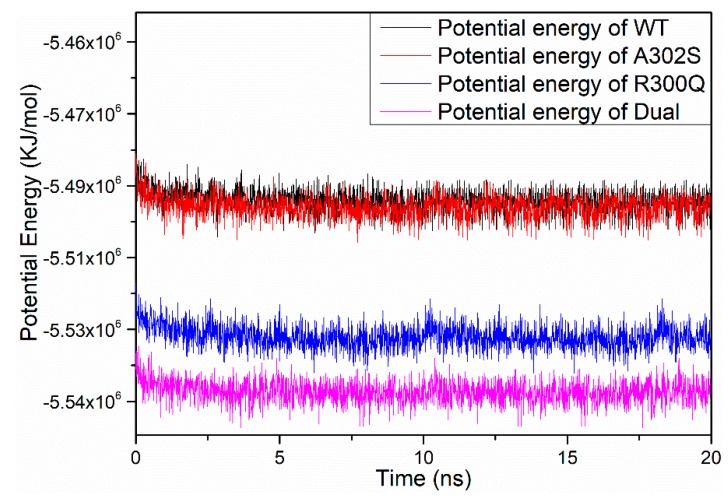
Potential energies of the wild-type (black), A302S (red), R300Q (blue), and dual-mutation (magenta) *N. lugens* RDLR models during the 20 ns molecular dynamics (MD) simulation.

**Figure 4:**
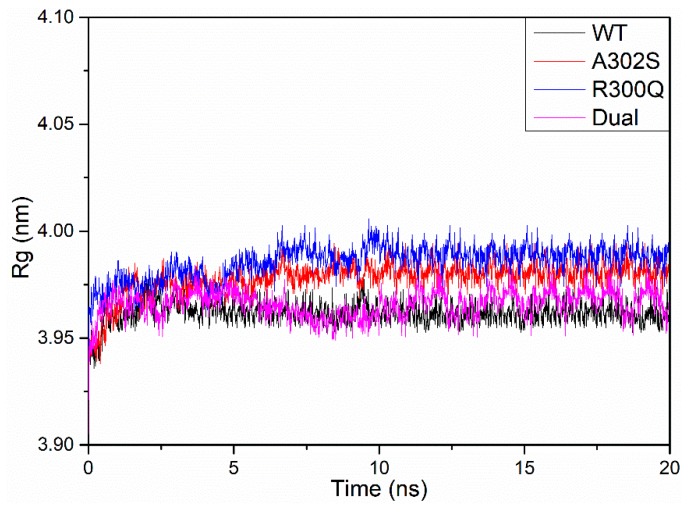
Gyration radii of the wild-type (black), A302S (red), R300Q (blue), and dual-mutation (magenta) *N. lugens* RDLR models during the 20 ns MD simulation.

**Figure 5:**
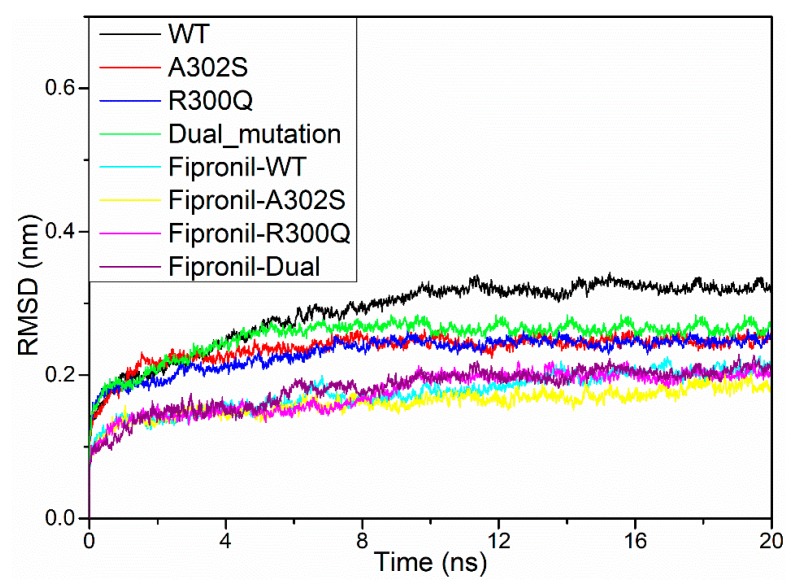
Root mean square deviation (RMSD) values of the backbone atoms of *N. lugens* RDLR models during the 20 ns MD simulation.

Figure 3 indicated that the average PE values of wild-type, A2′S, R0′Q, and dual-mutation models during 20 ns MD simulations that were maintained at −5.49 × 10^6^ KJ·mol^−1^, −5.50 × 10^6^ KJ·mol^−1^, −5.53 × 10^6^ KJ·mol^−1^, and −5.54 × 10^6^ KJ·mol^−1^, respectively, which suggested the stability of structures. As shown in Figure 4, it could be observed that the Rg of each model was equilibrated after an instant-time increase at the beginning of the simulations. Although the values of Rg had slight fluctuation, they were maintained at a constant rage, which also confirmed the stability of these structures. However, the Rg of R0′Q mutated RDLR model is slightly higher than those of the other RDLR models in the absence of A2′S mutation. Furthermore, the RMSD values (Figure 5) also ensured that the structures had been optimized to be stable. In conclusion, the structures of four models after 20 ns MD optimizations were suitable for the following study.

### 2.3. Ligand Docking

**Figure 6:**
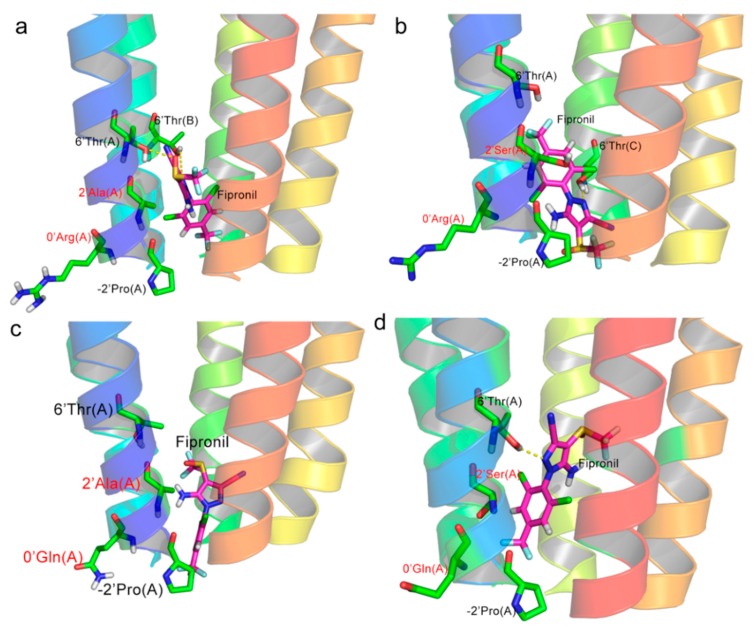
The binding mode of fipronil in binding pockets of different GABARs. (**a**) Fipronil-wild type (WT) GABAR, (**b**) Fipronil-A2′S GABAR, (**c**) Fiproinl-R0′Q GABAR, and (**d**) Fipronil-Dual mutant GABAR.

Fipronil was docked into four RDLRs, respectively, to investigate the binding interactions and modes. As depicted in Figure 6, similar binding poses of fipronil were observed in all three RDLR models, except A2′S RDLR; fipronil was erected in channel surrounded by five TM2 helices. When docked to the wild type (WT) RDLR model, the trifluoromethyl group of fipronil was oriented toward the intracellular domain, which is consistent with previous findings [26]. Interestingly, the conformation of fipronil was reversed, while 2′Ala was replaced by Ser; however, the conformation restored the same orientation as that in the WT RDLR model, while A2′S was accompanied with R0′Q. Different binding poses might have significant influence on resistance of fipronil. The N−H···O−H-bond between the amino group of fipronil and the side chain of 6′Thr could be observed in WT systems, which has been reported to be crucial for fipronil binding by other researchers [27,28,29]. Moreover, the residues 2′Ala and 9′Leu, which were reported to play important roles for fipronil binding in previously investigations [30,31,32], showed significant interactions in all docking results, which suggested the reliability of the modeled 3D structures of RDLRs and docking results.

CScore is an important scoring function for binding affinity prediction [33], which always reports the output of the docking energies as total score. CScore could be converted into binding free energy (ΔG_binding_ = −2.303RT × total score). Hence, the total score could reflect the binding ability between the receptor and the ligand. As shown in Table 2, the total score of fipronil-A2′S RDLR was slightly higher than that of fipronil-WT RDLR, and the total scores of fipronil-R0′Q and fipronil-dual RDLR were obviously lower, which indicated weaker interactions between fipronil with both RDLR s. The docking results revealed that, when amino acids were replaced in RDLRs, the interactions between receptors and ligands changed, and fipronil would show weakest interactions with RDLRs when the R0′Q mutation was accompanied with A2′S mutation. The docking results were almost consistent with the previous conclusions [19].

### 2.4. Molecular Dynamics Simulations

In MD studies, RMSD is a key parameter for evaluating the conformation stability of proteins and ligands. Figure 5 shows the RMSD values for ligands and the backbone atoms of proteins, which illustrated that all four complexes become stable gradually after 9 ns simulations. However, in the last 14 ns of the simulations, the amplitude of the equilibrium deviation was slightly different in each trajectory. The stability of the protein relative to its conformation can be determined by the deviations that were produced during the course of its simulation. Smaller the deviations always associate with more stable the protein structure. When bound with fipronil, the RDLRs were apparently more stable than the ones without fipronil. Moreover, the RMSD values of fipronil-R300Q and fipronil-dual complex were slightly higher than the other two systems, which might bethe consequence of higher resistance to fipronil.

**Figure 7:**
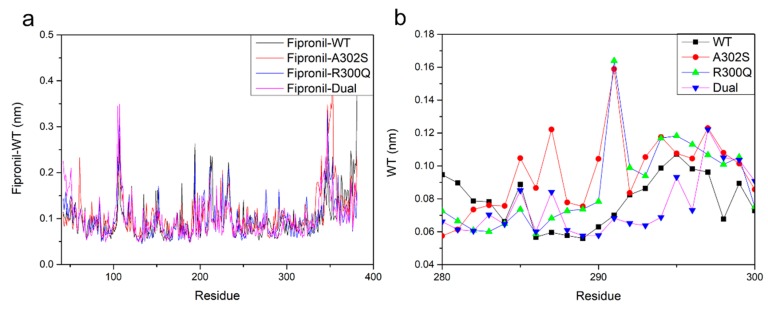
The root mean square fluctuation (RMSF) plots of GABAR (**a**) and TM2 residues (**b**) during the 10 ns MD simulation.

The root mean square fluctuation (RMSF) values were calculated to determine the flexibility and mobility of each residue during the MD simulations. As shown in Figure 7a, the RMSF values for the residues of TM domains were relatively low, which indicated the stability of TM domains during the MD simulation. However, the fluctuating residues with higher values were observed far from the ligand binding site, which implied that these residues might have a weak effect on the interaction of fipronil with RDLR. Figure 7b depicts RMSF versus residues plot in ligand binding pocket within the TM2 domain for detailed analysis. Obviously, when fipronil bound to dual-mutation GABAR, the RMSF values showed lower than the other three systems, which suggested that residues from fipronil-dual system might undergo smaller fluctuation as compared with the other three systems. The smaller fluctuation might be the consequence of high level of fipronil resistance.

Molecular mechanics Poisson–Boltzmann surface area (MM-PBSA) methods were applied to obtain binding free energies of fipronil in four models during the MD simulation to estimate the strength of interaction between fipronil and different mutant RDLRs. Table 3 indicated that the van der Waals interaction have more important contribution for the total interaction energies in all systems. The ΔG_binding_ values of all systems showed that the order of favorable binding interactions is as given: Fipronil-A302S (−161.355 KJ/mol) > Fipronil-WT (−140.868 KJ/mol) > Fipronil-R300Q (−118.729 KJ/mol) > Fipronil-Dual (−45.240 KJ/mol), which is consistent with the docking results. When R300Q mutation was associated with A302S mutation, the binding free energy of fipronil showed the lowest level, which revealed that fipronil has the weakest interaction with dual mutation RDLR model. Generally, the binding free energies of fipronil in four RDLRs are in agreement with the experimental results.

## 3. Materials and Methods

### 3.1. Protein Homology Modeling and Evaluation

Since the majority of experimental structure are not available, computational methods, such as homology modeling, are used to predict the structure and function of 3D protein models. The selection of template is of great significance during homology modeling, and the chosen of template could directly influence their quality and even determine the main folding of the target structures. The target sequence of *N. lugens* RDL (Uniprot ID: AGK30293) subunit was retrieved from the Uniprot database (http://www.uniprot.org/). The crystal structure of Human β3 GABA_A_R (PDB ID: 4COF, resolution: 2.97 Å) was selected as the template due to its relatively high resolution and high identity to RDL. The mature GABA_A_R β3 isoform 1 with the intracellular M3–M4 loop (Gly 333–Asn 446) replaced with the short amino acid sequence SQPARAA is the most suitable construct in terms of yield and monodispersity. Accordingly, the sequence from Gly308 to Asn421 in 4COF was replaced by the SQPARAA amino acid sequence [34]. Here, the same process was performed on the sequence of *N. lugens* RDL subunit in order to guarantee the accuracy of building subunits. MSA was performed while using ClustalW server [35], and the results were transformed for more intuitive comparison by ESPript 3.0 [36] on the basis of the MSA. At last, the 3D structure of *N. lugens* GABAR were constructed according to the MSA results by the three different tools, including SWISS-MODEL [37], Modeller 9.21, and SYBYL-X 2.1 (Tripos Inc., St Louis, MO, USA), respectively.

Programs including PROCHECK, ERRAT, and VERIFY 3D in the SAVES server (http://servicesn.mbi.ucla.edu/SAVES/) were employed to evaluate the qualities of the generated 3D structures and choose the best model for further study. Ramachandran plot acquired from the PROCHECK program could represent the phi and psi angles distribution of each residue in the protein. ERRAT is a quality factor for non-bonded atomic interactions. The generally accepted score range is >50 for a relatively reasonable model and the higher ERRAT is always associated with better quality. The verified 3D could show the percentage of the residues have averaged 3D-1D score ≥ 0.2. Additionally, ProSa server [38] (https://prosa.services.came.sbg.ac.at/prosa.php) was also applied to verify the qualities of structure. The energy evaluation of structure, as carried out by ProSa, could generate a Z-score distribution area, the lower Z-score, the higher quality.

### 3.2. Model Optimization

The protein models that were generated by homology modeling are usually unstable, for example, there would be unfavorable bond lengths, bond angles, and torsion angles. Therefore, it is essential to modify the model structures for further study. Four constructed RDLR models were conducted for 20 ns MD simulations while using GROMACS package 2016.05 with AMBER99SB force field to minimize the energy and obtain more stable structures. After the MD simulations, the potential energy and radius of gyration was calculated to assess the stability of the structure by the energy and gyration module, respectively. Besides, the RMS module also calculated the RMSD values. At last, the after-10ns MD structures were derived for the next study by trjconv module.

### 3.3. Docking Analysis

SYBYL-X 2.1 (Tripos Inc.) constructed the structure of fipronil. Then, the molecular was optimized with an energy gradient convergence criterion of 0.005 kcal/(mol·Å) and a maximum of 10,000 iterations. The other parameters were set to default values [24].

Docking simulations of fipronil into the binding pocket of *N. lugens* RDLRs were conducted by the surflex-dock module of SYBYL software in this study. The surflex-dock utilizes a so-called “whole” molecule alignment algorithm that was based on morphological similarity between the ligand and target [39]. The residues mode was adopted to generate the protomol in the Surflex-dock program, the −2′ and 2′ residues Mol Area from five chains were selected to generate protomol. In the docking run, 20 conformations of fipronil were obtained through Surflex-dock, and all of the conformations were extracted from the optimized fipronil-GABARs complexes. Among these conformations, one with the highest total scores would be selected as the original binding conformation for following MD simulation [40].

### 3.4. Molecular Dynamics Simulations

The MD simulations were performed while using GROMACS software package [41]. AMBER99SB force filed, which is recommended for biological macromolecules, such as proteins and nucleic acids, was used in this study. The relative files of topology and force field parameters files for fipronil were generated by ACPYPE [42], a Python tool that uses Antechamber to automatically generate topology files for small chemical compounds. After the preparation of relative files, all systems were put into a 20 ∙20 ∙20 Å^3^ cubic box with full of SPC water model, respectively. Subsequently, chloride ions were added into the boxes to neutralize the charge of system. Prior to the simulations, all of the systems conducted energy minimization in AMBER 99SB force field without constraints, while using the steepest descent integrator for 50,000 steps until a tolerance of 10 kJ mol^−1^. After being equilibrated at 300 K using V-rescale for 100 ps as NVT ensemble, the NPT ensemble followed for 10 ns at 1.02 bar using the Parrinello–Rahman algorithm [43]. Finally, MD simulations were carried out for 20 ns, respectively. During the simulations, the linear constraint solver algorithm was used to constrain the length of covalent bonds. Additionally, the particle-mesh Ewald summation technique was used to compute long-ranged electrostatic interactions. The coulomb and van der Waal’s cut-offs were set to 1.0 and 1.4 nm, respectively. The time step of trajectory was set to 2 fs and the coordinate trajectories were written at intervals of 10 ps. The binding free energies between RDLRs and fipronil were calculated by MM-PBSA (Molecular mechanics Poisson–Boltzmann surface area).

## 4. Conclusions

Three comparative methods were applied to build the 3D models of *N. lugens* RDLR while using Human β3 GABA_A_R as the template. The best model was chosen to generate the A2′S, R0′Q, and double-mutated (A2′S + R0′Q) models. After optimizing the structures, fipronil was then docked into these RDLRs. The docking results revealed that binding pose changed when mutations were conducted. Binding free energies that were calculated using MMPBSA method were consistent with the docking results. The results revealed when R0′Q mutation in combination with A2′S, fipronil showed weakest interactions with RDLRs, which is consistent with the experimental results. These obtained results may provide guidance for the development of novel insecticides and could help to realize the fipronil-resistance mechanism in insect RDLRs.

## Figures and Tables

**Table 1:** Evaluation assessment of the *N. lugens* RDLR models that constructed using different methods.

Methods	Ramachandran Plot ^a^		Z-Score ^b^	Percentage of the Residues have Averaged 3D-1D Score ≥ 0.2 ^c^	ERRAT (Overall Quality Factor) ^d^
	Allowed Regions	Disallowed Regions			
SWISS-MODEL	99.5%	0.5%	−4.85	70.61%	89.76
MODELLER	99.8%	0.2%	−4.74	70.25%	75.91
SYBYL	99.3%	0.7%	−3.16	73.35%	79.00

^a^ The phi and psi angles distribution of each residue in the protein. ^b^ Energy evaluation of structure, carried out by ProSa. ^c^ Assessment of protein models with 3D profiles. ^d^ Quality factor for non-bonded atomic interactions.

**Table 2:** Docking scores, hydrogen bonds, and binding free energies of the docked fipronil in WT and mutant RDLRs.

Model	Hydrogen Bonds	Total Score	ΔG_binding_ (KJ/mol)	IC_50_^a^
WT	6′Thr(A) with Fipronil	3.311	−18.901	19.81 ± 3.31
A302S	6′Thr(C) with Fipronil	3.59	−20.494	45.47 ± 7.05
R300Q	-	2.91	−16.612	96.36 ± 11.27
Dual-mutation	6′Thr(A) with Fipronil	2.21	−12.616	124.75 ± 16.03

^a^ Influence of mutations on fipronil sensitivity were retrieved from experimental results of Zhang et al. [18].

**Table 3:** The binding free energies (KJ/mol) of fipronil in *N. lugens* WT and mutant RDLRs calculated by the Molecular mechanics Poisson–Boltzmann surface area (MM-PBSA) method.

No.	ΔE_vdw_	ΔE_ele_	ΔG_PB_	ΔG_SA_	ΔG_binding_
Fipronil-WT	−169.155	−59.646	105.167	−17.233	−140.868
Fipronil-A302S	−187.221	−127.879	171.830	−18.084	−161.355
Fipronil-R300Q	−214.681	−50.407	165.273	−18.915	−118.729
Fipronil-Dual	−174.414	1.745	146.088	−18.659	−45.240

## Supplementary Materials

The following are available online. Figure S1: The Ramachandran plot of the constructed *N. lugens* RDLR model, Figure S2: The ProSA evaluation of the constructed *N. lugens* RDLR model, Table S1: Evaluation assessment of the template and mutant models.

## Abbreviations

GABA	γ-aminobutyric acid
GABAR	γ-aminobutyric acid receptor
CNS	Central nervous system
NCA	Noncompetitive antagonist
RDL	Resistant to dieldrin
WT	Wild type
MD	Molecular dynamics
3D	Three-dimension
PE	Potential energy
RDLR	RDL GABA receptors
Rg	Radius of gyration
RMSD	Root-mean-square deviation
RMSF	Root mean square fluctuation
MSA	Multiple sequence alignment
