# Supplementary material for: Identification of The Fipronil Resistance Associated Mutations in Nilaparvata lugens GABA Receptors by Molecular Modeling
Source: Molecules. 2019 Nov 14;24(22):4116. doi: 10.3390/molecules24224116 (PMC6891292; doi:10.3390/molecules24224116)
Supplement: Supplementary file 1 [file molecules-24-04116-s001.pdf]

molecules
MDPI

Supplementary Materials

Identification of The Fipronil Resistance Associated Mutations in *Nilaparvata lugens* GABA Receptors by Molecular Modeling

Yafeng Tian, Ya Gao, Yanming Chen, Genyan Liu * and Xiulian Ju *

Key Laboratory for Green Chemical Process of Ministry of Education, School of Chemical Engineering and Pharmacy, Wuhan Institute of Technology, Wuhan 430205, China; 21706010103@wit.edu.cn (Y.T.); gaoya_007@yeah.net (Y.G.); yanmingchen2018@163.com (Y.C.)

* Correspondence: liugenyan@wit.edu.cn (G.L.); xiulianju2001@yahoo.com (X.J.); Tel.: +86-27-8719-4980 (G.L.)

Table S1. Evaluation assessment of the template and mutant models.

Model	Ramachandran Plot [a]		Z-score [b]	Percentage of The Residues have Averaged 3D-1D Score ≥ 0.2 [c]	ERRAT (Overall Quality Factor) [d]
	Allowed Regions	Disallowed Regions			
4COF	99.7%	0.3%	-5.5	65.71%	96.76
Wild type	99.5%	0.5%	-4.85	70.61%	89.76
A2′S	99.5%	0.5%	-3.78	71.31%	89.25
R0′Q	99.5%	0.5%	-3.85	68.31%	89.54
A2′S + R0′Q	99.5%	0.5%	-3.79	69.08%	88.93

[a] The phi and psi angles distribution of each residue in the protein. [b] Energy evaluation of structure, carried out by ProSa. [c] Assessment of protein models with 3D profiles. [d] Quality factor for non-bonded atomic interactions.

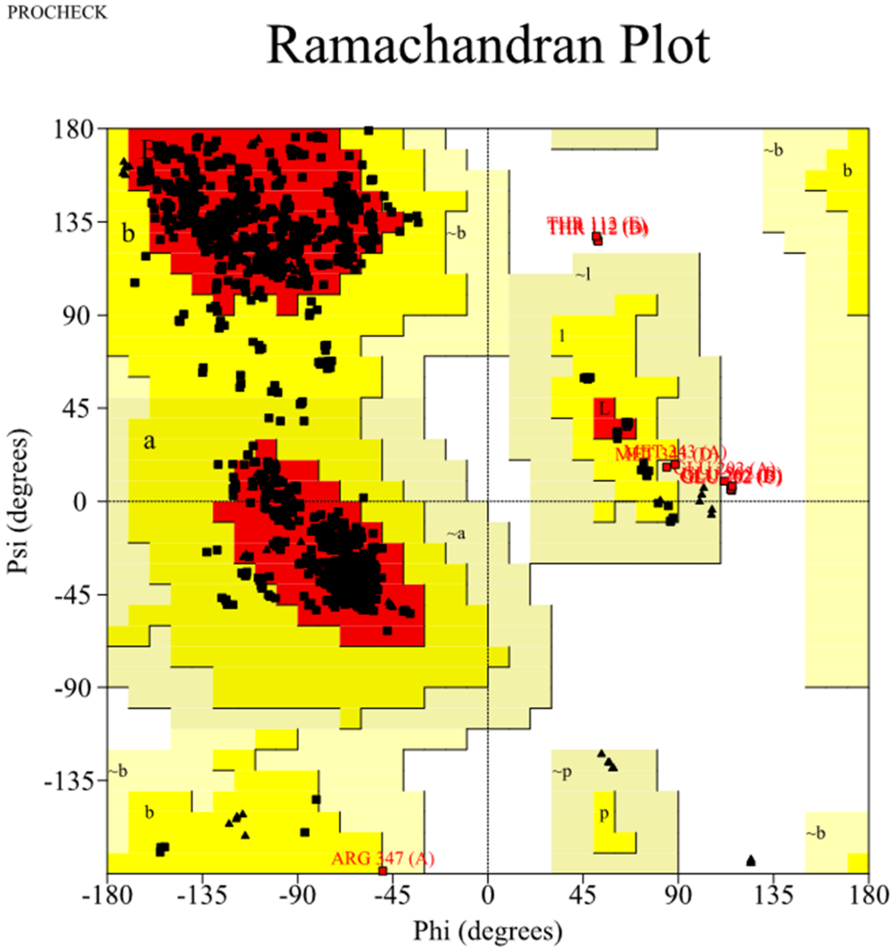

Plot statistics

Residues in most favoured regions [A,B,L]	1424	92.7%
Residues in additional allowed regions [a,b,l,p]	101	6.6%
Residues in generously allowed regions [~a,~b,~l,~p]	3	0.2%
Residues in disallowed regions	8	0.5%
	----	------
Number of non-glycine and non-proline residues	1536	100.0%
Number of end-residues (excl. Gly and Pro)	10	
Number of glycine residues (shown as triangles)	80	
Number of proline residues	75	

Total number of residues	1701	

Based on an analysis of 118 structures of resolution of at least 2.0 Angstroms and R-factor no greater than 20%, a good quality model would be expected to have over 90% in the most favoured regions.

Figure S1. The Ramachandran plot of the constructed *N. lugens* RDLR model.

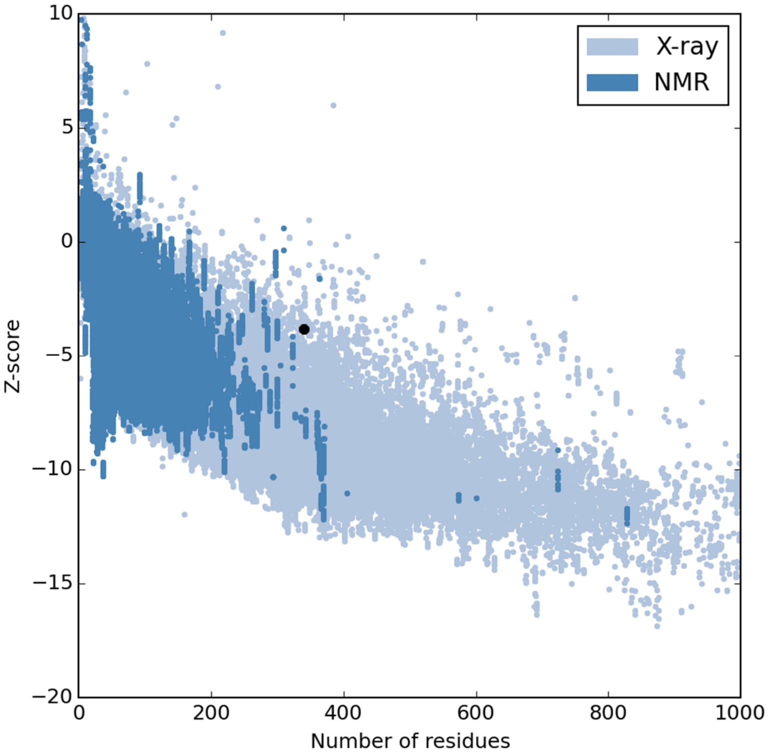

Figure S2. The ProSA evaluation of the constructed *N. lugens* RDLR model.
